# Antimicrobial resistance in *Staphylococcus pseudintermedius* on the environmental surfaces of a recently constructed veterinary hospital in Southern Thailand

**DOI:** 10.14202/vetworld.2022.1087-1096

**Published:** 2022-04-26

**Authors:** Punpichaya Fungwithaya, Narin Sontigun, Worakan Boonhoh, Kanpapat Boonchuay, Tuempong Wongtawan

**Affiliations:** 1 Centre of Excellence Research for Melioidosis and other Microorganism, Walailak University, Nakhon Si Thammarat 80160, Thailand; 2 Centre for One Health, Akkhraratchakumari Veterinary College, Walailak University, Nakhon Si Thammarat 80160, Thailand

**Keywords:** antimicrobial resistance, cleaning, methicillin-resistant *Staphylococcus pseudintermedius*, veterinary hospital

## Abstract

**Background and Aim:**

*Staphylococcus pseudintermedius* is a zoonotic bacterium commonly found in animals, especially dogs. These bacteria can survive on environmental surfaces for several months. The infection of *S. pseudintermedius* from the environment is possible, but properly cleaning surface objects can prevent it. This study aimed to investigate the prevalence of methicillin-resistant *S. pseudintermedius* (MRSP) in the environment of a recently constructed veterinary hospital in Southern Thailand, where we hypothesized that the prevalence of MRSP might be very low.

**Materials and Methods:**

At three different time points, 150 samples were collected from different environmental surfaces and wastewater across the veterinary hospital. The collection was done after the hospital’s cleaning. Bacteria were purified in the culture before being identified as species by biochemical tests and polymerase chain reaction (PCR). Next, the antimicrobial-resistant profile was performed using an automated system (Vitek 2). Finally, the antimicrobial resistance genes were identified using PCR.

**Results:**

Fifteen colonies of *S. pseudintermedius* were isolated from the surfaces of eight floors, four tables, two chairs, and one rebreathing tube. Fourteen colonies (93.3%) were multidrug-resistant (MDR) and carried the *blaZ* gene (93.3%). The majority of colonies were resistant to benzylpenicillin (93.3%), cefovecin (93.3%), ceftiofur (93.3%), kanamycin (93.3%), and neomycin (93.3%). Notably, only four colonies (26.7%) were methicillin-susceptible *S. pseudintermedius*, whereas 11 colonies (73.3%) were MRSP and carried both the *mecA* and *blaZ genes*. Five MRSP (45.5%) were resistant to at least 14 antimicrobial drugs, represented as extensively drug-resistant (XDR) bacteria. Ten of eleven MRSP (90.9%) were Staphylococcal chromosomal *mec* type V, while another displayed untypeable. Despite the routine and extensive cleaning with detergent and disinfectant, MRSP isolates were still detectable.

**Conclusion:**

Many isolates of MRSP were found in this veterinary hospital. Almost all of them were MDR, and nearly half were XDR, posing a threat to animals and humans. In addition, the current hospital cleaning procedure proved ineffective. Future research should be conducted to determine the bacterial biofilm properties and bacterial sensitivity to certain detergents and disinfectants.

## Introduction

*Staphylococcus pseudintermedius* is a common pathogen that can be found in the environment, particularly on the surfaces of veterinary hospitals [1,2] and houses [3]. This organism is an opportunistic bacterium that is frequently identified in many companion animals such as dogs, cats, avians, and rabbits [4,5]. The colonization of *S. pseudintermedius* on canine skin can be persistent, sporadic, or transient depending on the dos [3,6]. These bacteria can cause numerous diseases in dogs, such as pyoderma, otitis externa, reproductive tract infection, respiratory tract infection, and urinary tract infection [4,7]. Methicillin-resistant *S. pseudintermedius* (MRSP) can be chronic and severe, and bacteria are typically detected on the infected lesion after a week of antimicrobial treatment [6,8]. Moreover, MRSP is regularly transferred between dogs and the environment throughout the veterinary hospital, which includes a rebreathing circuit, clippers, and floors [2,3,9]. Other staphylococci, including *S. aureus*, can also be present on the surface of veterinary hospitals and are capable of transferring the resistome to *S. pseudintermedius* [10]. Although *S. pseudintermedius* is zoonotic, human infections are rare and mainly limited to bite wounds; a few cases of septicemia resulting in brain abscess, endocarditis, sinusitis, otitis, arthritis, and pneumonia are reported [11-14].

Globally, the incidence of MRSP has been increasingly reported [15]. This pathogen carries the *mecA* gene and the Staphylococcal chromosomal *mec* (SCC*mec*), a large mobile genetic element shares among staphylococci [1,16] and contains extensively drug-resistant (XDR) genes [17]. Many MRSP also carried the *blaZ* gene, which produces penicillinase capable of degrading beta-lactam antibiotic drugs [18]. Most MRSPs are multidrug-resistant (MDR), causing treatment difficult and threatening a dog’s life, but MRSP infection is uncommon in cats [4,7,19]. The transmission of resistant genes among staphylococci is one of the crucial factors in the emergence of antimicrobial XDR and MDR bacteria that are potentially detrimental to human health [20].

Although reports of MRSP in veterinary hospitals in Thailand are rare and limited to older hospitals in central Thailand, those investigations suggest that MRSP is frequently discovered on the hospital’s environmental surface [1,2,18]. Therefore, we hypothesized that the high prevalence of MRSP might be associated with the age of veterinary hospitals. This study aimed to determine the prevalence of MRSP on the environmental surface of a recently constructed veterinary hospital in southern Thailand, where we believed that the prevalence of MRSP might be very low. This study will provide the knowledge to understand the colonization site and antimicrobial profile of *S. pseudintermedius*, leading to sanitation, hygiene, and treatment plans.

## Materials and Methods

### Ethical approval

This study was approved by the Walailak University-Institutional Biosafety Committee (IBC) (WU-IBC-63-027).

### Study period and location

The samples were collected from December 2020 to April 2021. A study site was a veterinary hospital located in Nakhon Si Thammarat Province, Southern Thailand.

### Sample collection

This hospital started operations in October 2018 and was open daily from 8.30 AM to 4.30 PM, with an average of approximately ten animals treated per day. The building was two stories tall, with the first-floor housing medical rooms for post-operative care, vaccinations, and an intensive care unit. The second level was dedicated to the surgical unit, including a preparation room and two operating rooms. Daily routine cleaning was done after the hospital closed at 4.30 PM using detergent by the cleaning company.

The samples were collected by swabbing 50 different environmental surfaces across the hospital. Sample collections were done 3 times at different time points (1.5 months for each collection). The first sample collection was done after the routine cleaning; all surfaces, including floors, doors, tables, chairs, and equipment, were cleaned with the company’s detergent for 30 min in each room. For the second sample collection, all surfaces were cleaned with detergent and disinfectant (Dermodacyn Disinfecting Solution, CA, USA) twice in every room. Finally, the third sample collection occurred after the cleaning with detergent and disinfectant twice and later 30 min of fumigation (Dermodacyn Disinfecting Solution) in each room on the second floor, while the first floor was cleaned with detergent and disinfectant.

Each collection included 50 samples from frequent hand-touch locations and wastewater (before recycling). On the first floor, samples were taken from 17 floor surfaces, ten table surfaces, two chair surfaces, two doorknobs, one refrigerator handle, and two wastewater sites. On the second floor, 16 samples were collected from the preparation room and surgery room, eight samples from the floor, four samples from tables, and four samples from equipment.

Before sample collection, sterile cotton swabs were moistened with sterile tryptic soy broth (TSB) (Oxoid, Hampshire, UK). On the floor, surface swab sampling was performed at 1 cm^2^ per site, whereas on tables and equipment, surface swab sampling was performed by rolling a cotton bud around the surface [21]. Samples were kept on ice and then transported within a sealed box to a microbiology laboratory for identification within 2 h.

### Bacterial identification

Samples in TSB were incubated at 37°C for 24 h. Next, one suspension loop was placed on mannitol salt agar (Oxoid) supplemented with 0.5 μg/mL of oxacillin (Oxoid). Three staphylococcus-like colonies were picked and placed on blood agar for bacterial identification. Only colonies expressing hemolysin were chosen for biochemical identification using the VITEK 2 Compact, automated ID/AST instrument (Biomeriex, Marcy l’Etoile, France) and finally confirmed with multiplex polymerase chain reaction (PCR) as previously described by Sasaki *et al*. [22].

### PCR

For DNA extraction, the selected colony was put into 1 mL of TSB (Oxoid) and incubated at 37°C for 24 h. Bacterial DNA was extracted using Presto™ Mini gDNA Bacteria Kit (Geneaid, New Taipei City, Taiwan). DNA templates were used for species identification, the *mecA* gene detection, the *blaZ* gene detection, and the SCC*mec* detection as shown in Table-1 [22-25].

**Table 1:** The primer of Staphylococcal chromosomal mec typing.

Primer	Sequence (5’–3’)	Size of PCR product (bp)	References
*au-F3*	TCGCTTGCTATGATTGTGG	359	[22]
*au-R*	GCCAATGTTCTACCATAGC		
*in-F*	CATGTCATATTATTGCGAATGA	430	
*in-R3*	AGGACCATCACCATTGACATATTGAAACC		
*sch-F*	AATGGCTACAATGATAATCACTAA	526	
*sch-R*	CATATCTGTCTTTCGGCGCG		
*hy-F1*	CATTATATGATTTGAACGTG	793	
*hy-R1*	GAATCAATATCGTAAAGTTGC		
*pse-F2*	TRGGCAGTAGGATTCGTTAA	926	
*pse-R5*	CTTTTGTGCTYCMTTTTGG		
*mecA-F*	AAAATCGATGGTAAAGGTTGGC	532	[23]
*mecA-R*	AGTTCTGCAGTACCGGATTTGC		
*blaZ-F*	ACTTCAACACCTGCTGCTTTC	173	[24]
*blaZ-R*	TGACCACTTTTATCAGCAACC		
M-PCR 1 (for amplification of *ccr* gene complex with *mecA*)			[25]
*mA1*	GCTATCCACCCTCAAACAGG	286	
*mA2*	ACGTTGTAACCACCCCAAGA		
*α1*	AACCTATATCATCAATCAGTACGT	695	
*α2*	TAAAGGCATCAATGCACAAACACT	937	
*α3*	AGCTCAAAAGCAAGCAATAGAAT	1791	
*βc*	ATTGCCTTGATAATAGCCITCT	1287	
*α4.2*	GTATCAATGCACCAGAACTT		
*β4.2*	TTGCGACTCTCTTGGCGTTT		
*γR*	CGTCTATTACAAGATGTTAAGGATAAT	518	
*γF*	CCTTTATAGACTGGATTATTCAAAATAT		
M-PCR 2 (for amplification of *mec* gene complex class)			
*mI6*	CATAACTTCCCATTCTGCAGATG	1963	
*IS7*	ATGCTTAATGATAGCATCCGAATG	2827	
*IS2*	TGAGGTTATTCAGATATTTCGATGT		
*mA7*	ATATACCAAACCCGACAACTACA	804	

M-PCR=Multiplex polymerase chain reaction

PCR products were validated with electrophoresis on 1.5% agarose gel in 1x Tris-acid-EDTA buffer (Vivantis, Selangor Darul Ehsan, Malaysia) at 135 V/cm for 20 min. The DNA bands were visualized under UV light with the G-BOX F3 Gel imaging machine (Syngene, Cambridge, UK).

### Antimicrobial-resistant profile

All *mecA*-positive isolates were examined for antimicrobial resistance (AMR) profiles using the AST-GP80 card with e the VITEK 2 Compact system based on minimal inhibitory concentration (MIC). Sixteen antimicrobial drugs were tested, including benzylpenicillin, cefovecin, ceftiofur, gentamicin, kanamycin, neomycin, enrofloxacin, marbofloxacin, pradofloxacin, erythromycin, clindamycin, doxycycline, tetracycline, nitrofuran, chloramphenicol, and trimethoprim/sulfamethoxazole. Most of the antimicrobial drugs in this study were routinely used in this hospital. The MIC was determined using VET: Clinical and Laboratory Standards Institute-based plus natural resistance data provided by the VITEK 2 Compact machine.

### Statistical analysis

A descriptive analysis of the defining bacteria distribution variable in this hospital was performed using Jamovi software version 2.0 [26]. The population of pathogens and XDR bacteria were described by percentile. MDR, extensively XDR, and pan drug-resistance (PDR) bacteria were categorized according to Magiorakos *et al*. [27]. The SCC*mec* types were interpreted as I to V types or untypable [25].

## Results

### Isolation

In total, bacteria were observed on 144 of 150 swabs (96%). Bacteria were detected in 31 of 50 samples (62.0%) from the first sample collection, 18 samples (36.0%) from the second sample collection, and 21 samples from the third sample collection (42.0%).

Specifically, 110 staphylococci-like colonies (73.3%) from 150 environmental samples were found on mannitol salt agar. Then 52 of 110 staphylococci-like colonies (47.3%) were confirmed as staphylococci by PCR. However, staphylococci were not detected in various areas, including dive floors, one stethoscope, one surgery equipment, one doorknob, and two wastewaters throughout three sample collections.

**Figure-1:**
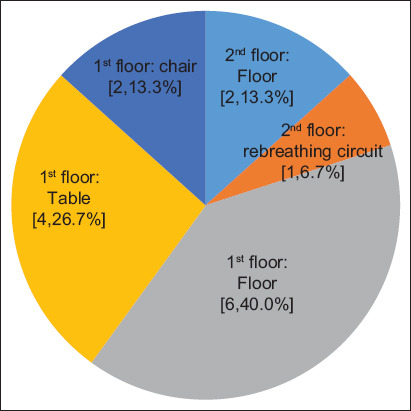
The prevalence of *Staphylococcus pseudintermedius* collected from the different environments in the veterinary hospitals. The numbers in the brackets represent the number of bacterial isolates and their percentage.

Fifteen colonies from 150 environmental samples (10.0%) and 52 staphylococci isolates (28.8%) were verified by PCR as *S. pseudintermedius*. The prevalence of *S. pseudintermedius* in the veterinary hospital environment is shown in Figure-1. Most of the *S. pseudintermedius* isolates were found on the first floors (12/15, 80.0%), and the majority of isolates were obtained from the floor surface (8/15, 53.3%). The remaining were found on the surfaces of chairs, tables, and a rebreathing circuit.

### MRSP

The data for MRSP and methicillin-susceptible *S. pseudintermedius* (MSSP) are shown in Table-2. Most colonies were MRSP (11/15, 73.3%), while the minority were MSSP (4/15, 26.7%). Most MRSP was detected on the floors (5/11, 45.5%), then tables (4/11, 36.4%), and waiting chairs (2/11, 18.2%), respectively. All MRSP colonies (positive for the *mecA* gene) were also positive for the *blaZ* gene. In addition, most MRSP isolates carried the SCC*mec* type V (10/11, 90.9%), while another displayed an untypable (NT) type.

**Table 2:** The number of *Staphylococcus pseudintermedius* colonies discovered during each collection period.

Sample site	1^st^ collection (Detergent)		2^nd^ collection (Disinfectant)		3^rd^ collection (Disinfectant)	
		
	MSSP	MRSP	MSSP	MRSP	MSSP	MRSP
Second floor	2					1
First floor			1		1	10

*Wiping and fumigation with disinfectant, MSSP=Methicillin-susceptible *Staphylococcus pseudintermedius*, MRSP=Methicillin-resistant *Staphylococcus pseudintermedius*

### Antimicrobial-resistant profile

The AMR profile is shown in Table-2. The highest resistant drugs were benzylpenicillin, cefovecin, ceftiofur, kanamycin, and neomycin (14/15, 93.33%), then erythromycin, tetracycline, and gentamycin (13/15, 86.7%). Conversely, the antimicrobial drugs with the highest sensitive drugs were nitrofurantoin (15/15, 100%), then chloramphenicol (13/15, 86.67%), and clindamycin (12/15, 80.0%), respectively.

### MDR

Fourteen colonies (93.3%) were MDR bacteria, including 11 MRSP and 3 MSSP colonies (Table-3). Five extensively XDR bacteria (33.3%) were found, including 4 MRSP colonies resistant to 15 antimicrobial drugs and one MSSP colony resistant to 14 drugs. All 11 MRSP colonies were MDR, with 36.4% (4/11) being XDR, whereas 75.0% (3/4) of MSSP colonies were MDR, with 25.0% (1/4) being XDR.

**Table 3:** Collection places, MRSP identification, *blaZ* detection, and antimicrobial-resistant profiles of *Staphylococcus pseudintermedius* in this veterinary hospital.

Places	MRSP (mecA)	*BlaZ* positive	Benzylpenicillin	Cefovecin	Ceftiofur	Gentamicin	Kanamycin	Neomycin	Enrofloxacin	Marbofloxacin
Floor in surgery room	MSSP	POS	R	R	R	R	R	R	S	S
Rebreathing circuit	MSSP	POS	R	R	R	R	R	R	S	R
Floor in preparation room	MRSP	POS	R	R	R	R	R	R	R	R
Floor in medication room 2	MSSP	POS	R	R	R	R	R	R	R	R
Floor in medication room 2	MRSP	POS	R	R	R	R	R	R	R	R
Examination table in medication room 2	MRSP	POS	R	R	R	R	R	R	R	R
Medication table in medication room 2	MRSP	POS	R	R	R	R	R	R	R	R
Equipment table in medication room 2	MRSP	POS	R	R	R	R	R	R	R	R
Floor in medication room 3	MRSP	POS	R	R	R	S	R	S	R	R
Floor in medication room 3	MSSP	NEG	S	S	S	S	S	R	S	S
Medication table in medication room 3	MRSP	POS	R	R	R	R	R	R	R	R
Floor in admission room 1	MRSP	POS	R	R	R	R	R	R	R	R
Floor in admission room 1	MRSP	POS	R	R	R	R	R	R	R	R
Waiting chair 1	MRSP	POS	R	R	R	R	R	R	R	R
Waiting chair 2	MRSP	POS	R	R	R	R	R	R	R	R
Percent of resistant results (%)			93.3	93.3	93.3	86.7	93.3	93.3	93.3	93.3

**Places**	**Pradofloxacin**	**Inducible clindamycin resistance**	**Erythromycin**	**Clindamycin**	**Doxycycline**	**Tetracycline**	**Nitrofurantoin**	**Chloramphenicol**	**trimethoprim sulfamethoxazole**	

Floor in surgery room	S	NEG	S	S	S	R	S	S	S	
Rebreathing circuit	S	NEG	S	S	S	R	S	S	S	
Floor in preparation room	R	NEG	R	R	S	S	S	S	R	
Floor in medication room 2	R	POS	R	S	R	R	S	S	R	
Floor in medication room 2	R	POS	R	S	R	R	S	S	R	
Examination table in medication room 2	R	POS	R	S	R	R	S	S	R	
Medication table in medication room 2	R	POS	S	S	R	R	S	S	R	
Equipment table in medication room 2	R	POS	R	S	R	R	S	S	R	
Floor in medication room 3	R	NEG	R	S	R	R	S	R	R	
Floor in medication room 3	S	NEG	S	S	S	S	S	S	S	
Medication table in medication room 3	R	NEG	R	R	R	R	S	S	R	
Floor in admission room 1	R	POS	R	S	R	R	S	S	R	
Floor in admission room 1	R	NEG	R	S	R	R	S	R	R	
Waiting chair 1	R	POS	R	S	R	R	S	S	R	
Waiting chair 2	R	NEG	R	R	R	R	S	S	R	
Percent of resistant results (%)	93.3		86.7	0.2	73.3	86.7	0	13.3	0.8	

MSSP = Methicillin-susceptible *Staphylococcus pseudintermedius*, MRSP = Methicillin-resistant *Staphylococcus pseudintermedius*

### Relation of cleaning and *S. pseudintermedius*

At the first collection time (cleaning with detergent), 2 MSSP colonies were discovered on the second floor, and they were positive for the *blaZ* gene. Then, for the second sample collection (after cleaning with detergent and disinfectant), only 1 MSSP colony without the *blaZ* gene was found on the first floor. For the final collection, 1 MRSP colony was displayed on the second floor (after cleaning with detergent, disinfectant, and fumigation); unexpectedly, many colonies (10 MRSP and 1 MSSP) were found on the first floor (after cleaning with detergent and disinfectant) (Table-2).

## Discussion

This study demonstrated that most areas (73%) of the recently constructed veterinary hospital could harbor *S. pseudintermedius*. The majority of colonies were MRSP and MDR, although the cleaning was done using a variety of methods. Almost all MRSP isolates carried the *mecA* and *blaZ* genes, as well as SCC*mec* type V.

The environment plays an important role in maintaining and transmitting staphylococci between animals and humans [28,29]. In the present study, the majority of MRSP isolates were discovered on frequent contact areas such as floors and veterinary tables, which is consistent with the previous studies [30,31]. Seriously, two colonies of MSSP were obtained from the surgical environment in the present study, although this environment is expected to be sterile to prevent surgical site infections. Staphylococci contamination of the surgical environment is occasionally found in veterinary hospitals and may cause surgical site infections [2,32]. Fortunately, *S. pseudintermedius* infection was not observed in any of the dogs operated in this hospital (unpublished data).

It has been noted that *Staphylococci* can remain on these environmental surfaces for at least 4 months before causing infection and the development of methicillin-resistant staphylococci (MRS) [33-36]. During hospitalization, dogs may come into contact with MRS, and the transmission between dogs, humans, and the environment can result in hospital- and community-acquired infections [37,38]. Moreover, the long-term circulation or persistence of *S. pseudintermedius* in pets and the environment can increase the probability of resistant genes being transmitted to other staphylococci, including *S. aureus*, an important pathogen in humans [10,37,38].

In this study, most colonies of *S. pseudintermedius* were MDR bacteria. They were resistant to several drug groups, such as beta-lactams, aminoglycosides, and fluoroquinolones. These groups of antimicrobial drugs are also commonly used in this veterinary hospital (e.g., amoxicillin/clavulanic acid, cefalexin, and enrofloxacin) and other hospitals [39-41]. The beta-lactam drugs, including penicillin, cefovecin, and ceftiofur, are the most commonly used antimicrobial drugs worldwide and in Thailand [1,8,42], and bacteria can be resistant to these drugs via several genes, such as the *mecA* and *blaZ* genes [43]. The co-existence of the *mecA* and *blaZ* genes was frequently observed in this study and other studies [15,43]; both genes can enhance bacterial AMR. However, no evidence of infection caused by *S. pseudintermedius* has been found in this hospital to date (personal communication). In the event of a future *S. pseudintermedius* infection, beta-lactamase inhibitors (to inhibit bacterial beta-lactamase) and nitrofurantoin (the most sensitive drug in this study) are recommended for therapy.

Surprisingly, many MRSP isolates were found on the first floor in this study, although the floor was cleaned with detergent and disinfectant. The disinfectant solution employed in this investigation contained 0.002% sodium hypochlorite and 0.013% hypochlorous acid, both of which have been shown in several papers to be effective against staphylococci when used as a liquid solution for *in vitro* studies [44-46]. Conversely, several reports argue that sodium hypochlorite does not effectively destroy staphylococci in the hospital environment [47-50], which might result from its instability after 24 h [48]. Another study shows that hypochlorous acid is inferior to chlorine dioxide as a disinfectant for hospital environments [50]. The conflicting results for hypochlorite as a disinfectant may be due to the varied quantities employed in each experiment, as many bacteria are eradicated at high concentrations (>0.5%) of sodium hypochlorite, including MRSA in human hospitals and MRSP in a veterinary hospital [49,51,52]. In this study, the disinfectant concentration might be lower than the recommendation [52], and hypochlorite and hypochlorous acid are not recommended for fumigation for eradicating bacteria. For fumigation, the theoretical options for sporicidal fumigants are formaldehyde, ethylene oxide, methyl bromide, hydrogen peroxide vapor, and chlorine dioxide [53]. Formaldehyde, in particular has been used to reduce bacteria in the surgery room [54]. Taking this together, using the high concentration of hypochlorite and hypochlorous acid as a liquid disinfectant might be recommended for cleaning this veterinary hospital [52].

Limited research has been conducted on the antibacterial efficacy of detergent or disinfectant products against *S. pseudintermedius* [52,55,56]. MRSP contamination in the environment can be reduced using household cleaning detergent and bleach, as well as antimicrobial treatment in infected dogs [55]. Furthermore, combining a high dose of chlorhexidine digluconate with cathelicidin (antimicrobial peptide) and incubating for 30-60 min can neutralize *S. pseudintermedius* [56]. A recent study in Thailand revealed that most MRSP obtained from dogs admitted to the veterinary hospital were strong biofilm producers [18]. It is possible that MRSP in this study could have the ability to form biofilms and develop resistance to the disinfectant. Further studies, such as biofilm formation assay, testing the effectiveness of detergents and disinfectants, must be done in the future to identify the cause of the existence of MRSP in the hospital and verify the most effective chemicals to eradicate it. Furthermore, the United States Environmental Protection Agency (https://www.epa.gov) has certified a number of commercial products as effective agents against MRSA; still, these products must be validated for their ability to eradicate MRSP.

Interestingly, the population of MRSP at a newly built veterinary hospital in southern Thailand was substantially lower than in prior findings in central Thailand, where the institution was almost 20 years old [1]. An older facility may be more susceptible to bacteria and antimicrobial drug accumulation than a new hospital. In addition, many factors may be involved in emerging antimicrobial-resistant bacteria, such as the number of patients, staff, location, and cleaning management.

## Conclusion

The present study demonstrated the presence of MDR MRSP in a newly constructed veterinary hospital, with the floors and veterinary tables being the most contaminated regions. MRSP appears to be resistant to the detergent and disinfectant used in this hospital. The limitation of this study is the connection between the pathogen, animal patient, owner, and hospital staff. In addition, the mechanism by which *S. pseudintermedius* tolerates detergents and disinfectants should be studied in the future, as well as the efficacy of various detergents and disinfectants against is pathogen.

## Authors’ Contributions

PF: Conception and designed the study, laboratory works, data analysis, and drafted the manuscript. NS and WB: Sample collection. KB: Laboratory works. TW: Conception, supervised the study, and revised manuscript. All authors have read and approved the final manuscript.
